# 2495. Inpatient Hepatitis C Virus Testing Increased During the COVID-19 Pandemic

**DOI:** 10.1093/ofid/ofad500.2113

**Published:** 2023-11-27

**Authors:** Brendan J Fitzgerald, Mamta K Jain, A L exander P Radunsky, Lauren N Cooper, Laura Hansen

**Affiliations:** UT Southwestern Medical Center, Dallas, Texas; UT Southwestern Medical Center, Dallas, Texas; Ut Southwestern, Dallas, Texas; University of Texas Southwestern Medical Center, Dallas, Texas; UT Southwestern, Dallas, TX

## Abstract

**Background:**

We have observed an unexpected increase in hepatitis C virus (HCV) testing in the inpatient setting since the beginning of the COVID-19 pandemic. We hypothesized that this increase was driven by abnormal liver function tests (LFTs) due to COVID-19. We undertook a large observational study to test this hypothesis and to better understand the predictors of inpatient HCV testing.

**Methods:**

We obtained medical record data for inpatients at Parkland Health and Hospital System (PHHS) from either of two 18-month time periods, representing before (8/1/2018-1/31/2020) and during (3/1/2020-8/31/2021) the COVID-19 pandemic. We evaluated possible predictors of inpatient HCV testing, including LFTs and COVID-19.

**Results:**

Among the 34940 patients examined (18170 pre-pandemic [PP] and 16770 during pandemic [DP]), those hospitalized DP were more likely to be Hispanic and to have charity coverage (Figure 1). DP, 14.8% of inpatients received an HCV antibody (Ab) test, as compared to 13.4% of inpatients PP (p< 0.001). This increase specifically occurred among patients with COVID-19, of whom 18.5% received an HCV Ab test (Figure 2). This finding correlated with a much higher occurrence of elevated initial LFTs among patients with COVID-19 (Figure 3). The positivity rate of inpatient HCV Ab tests decreased from 9.07% PP to 4.87% among patients with COVID-19 (p=0.002), but there was no difference between time periods for patients without COVID-19 (Figure 2). When considering the subset of patients with COVID-19 who had an abnormal LFT, the Ab positivity rate was 4.13% (95% CI = [2.15%, 6.12%]). Among these Ab-positive patients, the HCV RNA positivity rate was 31.3% (95% CI = [8.5%, 54.0%]).

**Figure:**
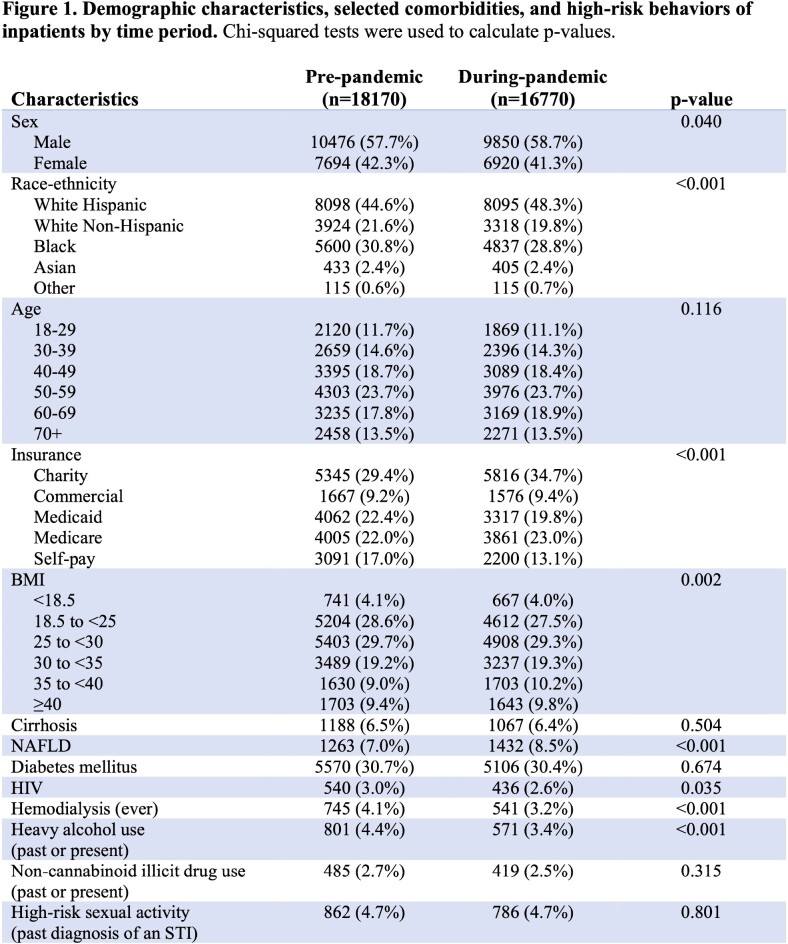

**Figure:**
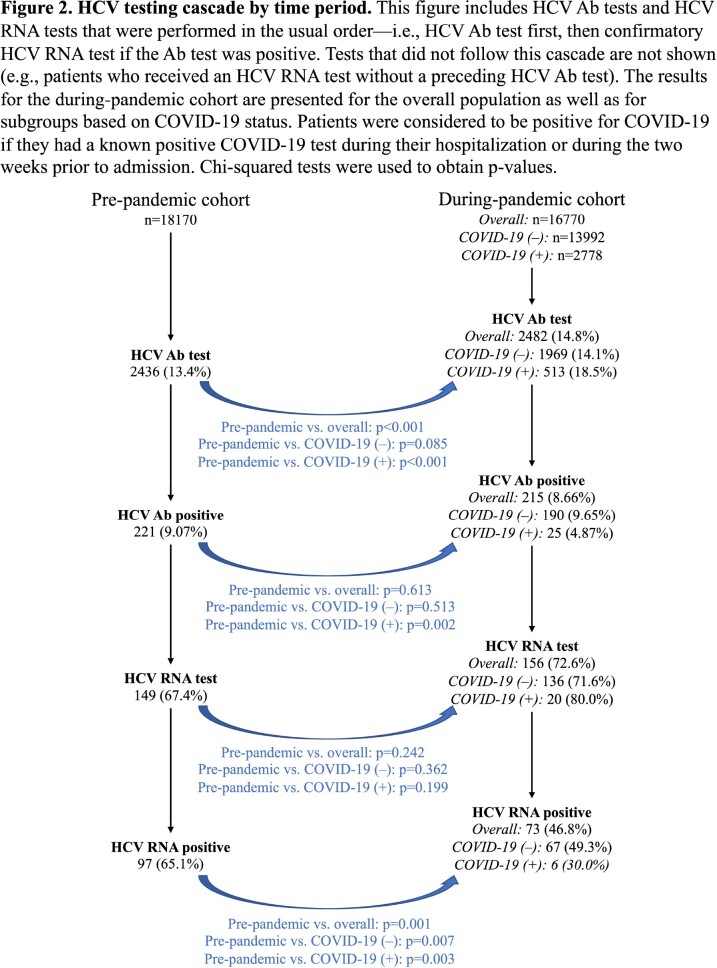

**Figure:**
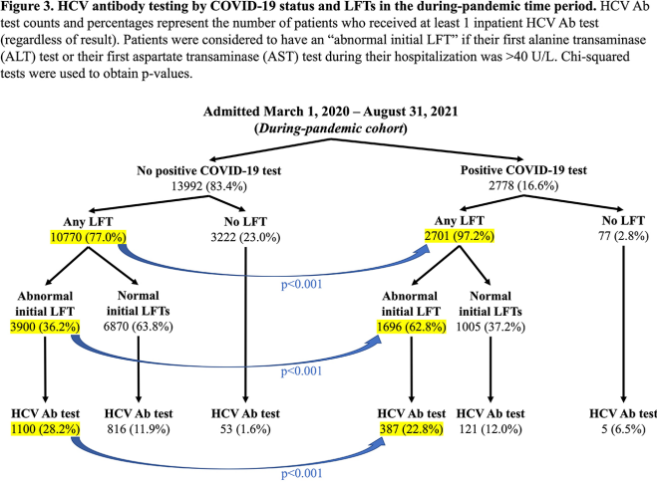

**Conclusion:**

Hepatic injury associated with COVID-19 inadvertently resulted in more HCV Ab testing among inpatients. The Ab positivity rate and RNA detection were lower among these patients. Compared to non-COVID-19 inpatients who have abnormal LFTs, those with COVID-19 represent a lower risk population for HCV. Nevertheless, HCV testing still led to detection of new cases. Although abnormal LFTs among those with COVID-19 likely reflect hepatic injury due to SARS-CoV-2, it should be considered as an opportunity to screen a population who may not otherwise receive routine healthcare.

**Disclosures:**

**Brendan J. Fitzgerald**, Pfizer Inc.: Stocks/Bonds|Viatris Inc.: Stocks/Bonds **Mamta K. Jain, MD, MPH**, Gilead Sciences: Grant/Research Support|Laurent: Grant/Research Support

